# The Apple Watch for Monitoring Mental Health–Related Physiological Symptoms: Literature Review

**DOI:** 10.2196/37354

**Published:** 2022-09-07

**Authors:** Gough Yumu Lui, Dervla Loughnane, Caitlin Polley, Titus Jayarathna, Paul P Breen

**Affiliations:** 1 The MARCS Institute for Brain, Behaviour and Development Western Sydney University Penrith, NSW Australia; 2 Virtual Psychologist Southport Park, QLD Australia; 3 Electrical and Electronic Engineering School of Engineering, Design and Built Environment Western Sydney University Penrith, NSW Australia; 4 Translational Health Research Institute Western Sydney University Penrith, NSW Australia

**Keywords:** Apple Watch, data, validation, mental health, psychology, precision medicine, heart rate variability, energy expenditure, sleep tracking, digital health, mobile phone

## Abstract

**Background:**

An anticipated surge in mental health service demand related to COVID-19 has motivated the use of novel methods of care to meet demand, given workforce limitations. Digital health technologies in the form of self-tracking technology have been identified as a potential avenue, provided sufficient evidence exists to support their effectiveness in mental health contexts.

**Objective:**

This literature review aims to identify current and potential physiological or physiologically related monitoring capabilities of the Apple Watch relevant to mental health monitoring and examine the accuracy and validation status of these measures and their implications for mental health treatment.

**Methods:**

A literature review was conducted from June 2021 to July 2021 of both published and gray literature pertaining to the Apple Watch, mental health, and physiology. The literature review identified studies validating the sensor capabilities of the Apple Watch.

**Results:**

A total of 5583 paper titles were identified, with 115 (2.06%) reviewed in full. Of these 115 papers, 19 (16.5%) were related to Apple Watch validation or comparison studies. Most studies showed that the Apple Watch could measure heart rate acceptably with increased errors in case of movement. Accurate energy expenditure measurements are difficult for most wearables, with the Apple Watch generally providing the best results compared with peers, despite overestimation. Heart rate variability measurements were found to have gaps in data but were able to detect mild mental stress. Activity monitoring with step counting showed good agreement, although wheelchair use was found to be prone to overestimation and poor performance on overground tasks. Atrial fibrillation detection showed mixed results, in part because of a high inconclusive result rate, but may be useful for ongoing monitoring. No studies recorded validation of the Sleep app feature; however, accelerometer-based sleep monitoring showed high accuracy and sensitivity in detecting sleep.

**Conclusions:**

The results are encouraging regarding the application of the Apple Watch in mental health, particularly as heart rate variability is a key indicator of changes in both physical and emotional states. Particular benefits may be derived through avoidance of recall bias and collection of supporting ecological context data. However, a lack of methodologically robust and replicated evidence of user benefit, a supportive health economic analysis, and concerns about personal health information remain key factors that must be addressed to enable broader uptake.

## Introduction

### Background

The COVID-19 pandemic has caused disruptions to the way people go about their daily lives. From the changing nature of work and employment, economic factors, the isolation brought about by stay-at-home orders, and the uncertainty of ever-changing health advice and medical directives, it is anticipated that these stresses will lead to an increase in mental health service demand beyond the current capacity [1]. The adoption of digital health technologies can potentially alleviate this burden.

Wearable devices are electronic sensors that are designed to be placed onto, or near to, the skin to measure signals from the body. Such devices can include wrist-worn devices similar to a watch or wristband, which can pair wirelessly with a mobile phone. Such devices have become a popular behavioral intervention for monitoring physiological activity to promote a healthy lifestyle [2]. Early forms of health monitoring include pedometers that would track daily steps and derive basic energy expenditure (EE) [3]. The potential of wearable devices for the monitoring of health has become particularly attractive to health care innovators seeking to enable new models of telehealth. However, these devices monitor physiological signals or physiologically related proxies (such as physical activity) of the user rather than mental health. Such devices may take the form of fitness trackers, which are typically simpler, lower-cost, and fixed-function devices with limited capabilities. Such devices often cannot support third-party apps, have limited user interactivity, and focus on fitness monitoring as their primary goal. By contrast, smartwatches are usually higher-end devices with a richer mix of sensors and user interfaces and a flexible, extensible software architecture permitting third-party software access and extended features such as voice calling, media control, and messaging. As the market matures, there are some products that may blur the lines; however, it is the richer suite of sensors, user interfaces, and support for third-party apps and data access, which makes these devices attractive for mental health research and monitoring purposes.

Mental health can be defined as “a state of wellbeing in which the individual realizes his or her own abilities, can cope with the normal stresses of life, can work productively and fruitfully, and is able to make a contribution to his or her community” [4]. This state is intimately connected with physical health and forms an integral part of general or overall health [5]. A mediation study examined the effects of physical health on mental health and vice versa, finding significant direct and indirect effects and cross-effects [6]. Studies have also indicated the effectiveness of physical activity in improving anxiety and depressive symptoms [7]. The measurement of signals from wearable devices that allow for an understanding of physical activity may also allow mental health status to be inferred.

### Motivation

Apple Inc has emerged as an industry leader in health technology and wellness tracking devices [8]. The Apple Watch, first introduced in 2015, has retained the largest market share since its introduction and has continually advanced the capabilities of smartwatches [9]. These devices are primarily intended as wellness tools, garnering additional personal health monitoring for the wearer, typically for physiological activities such as heart rate (HR), HR variability (HRV), respiration rate, and physiologically related measures such as EE and fall detection. Some capabilities of these devices, such as the electrocardiogram (ECG) function, including a supporting app, have received Food and Drug Administration (FDA) clearance [10], whereas other aspects of their sensors and app capabilities have not yet been independently validated or received regulatory clearances. Monitoring of stress using these devices has been less studied but appears to be a promising avenue for application, particularly in the mental health sphere.

As digital health provides a novel model of care through the use of intelligent data, computing, and telecommunications, it holds promise for meeting the challenges of increased mental health demands. It can also enable *precision medicine*, which provides treatments bespoke to the patient’s needs [11]. There is interest in digital health across a number of industry sectors, including health care providers, insurers, and businesses [12-17], that may desire access to information on personal health through wearable devices such as the Apple Watch.

Wider adoption of devices for mental health monitoring is, in part, hampered by a lack of clarity regarding the devices’ capabilities, the accuracy and validity of the data that are collected, and their applicability to mental health monitoring and diagnosis [18-20]. This research aimed to fill this knowledge gap by examining the embedded sensor capabilities within the Apple Watch range, the physiological and physiologically related metrics recorded and made available for analysis, the validation status of these metrics within the literature, the connections (where they exist) between relevant health conditions associated with each metric, and implications for treatment. This analysis was performed both in a “top-down” approach focusing on reviewing published literature regarding the Apple Watch and a “bottom-up” approach focusing on the hardware and software capabilities of the Apple Watch to identify both currently available features and potential features that could be operationalized through the creation of customized apps using the Apple WatchKit, CareKit, and ResearchKit frameworks.

## Methods

The literature review was conducted from June 2021 to July 2021.

### Types of Studies and Materials

Various types of published studies and editorials were included. The types of studies were extended to some unpublished (gray) literature that was evaluated and reviewed for its suitability to close gaps in knowledge. Other gray literature sources included developer documentation for the HealthKit application programming interface for storing and managing data collected on the devices. Several opinion pieces were reviewed contextually to further provide a professionally informed perspective or illustrate further points of consideration. This literature review was structured to include the literature concerning the monitoring of physical conditions that may present with psychological stressors and the implementation of the Apple Watch for such monitoring.

### Search Strategy

The electronic databases selected for this literature review were PubMed, Scopus, and Google Scholar. A list of secondary keywords (Textbox 1) was developed with an emphasis on “Apple Watch” and truncated keywords combined using Boolean operators. Publication dates were restricted to 2015 onward, coinciding with the announcement of the first Apple Watch. Other recent literature that included wearable devices and novel developments to monitor or detect depression, anxiety, or stress was also included in the search process, in addition to reviews and systematic reviews.

**Literature review secondary search terms.**

**Secondary search terms**

- “anxiety”
- “atrial fibrillation”
- “collection”
- “data”
- “depression”
- “digital health”
- “heart rate*”
- “insomnia”
- “mHealth”
- “monitor*”
- “oximet*”
- “physiology*”
- “psychology*”
- “remote”
- “respiration rate”
- “sens*”
- “sleep”
- “sleep apn*”
- “stress”
- “telehealth”
- “validat*”
- “wearable”

### Selection Process

Published literature was included based on its use of the Apple Watch for either physiological data validation or psychology or mental health studies. Areas of interest for applications in monitoring physiological stress and mental health included HR monitoring, sleep tracking, respiration monitoring, and EE. Other inclusion criteria included studies performed on the suitability of wearable devices for monitoring physiological stress and their impacts on mental health. Only publications in English were included in the review. Screening was performed by a primary researcher and reviewed by other authors. Duplicate studies were removed.

### Data Collection Process

Data extraction was performed using a spreadsheet that synthesized the findings and grouped the studies. Data management was achieved using EndNote (Clarivate Analytics) as the bibliographic management software. Where studies did not specify the Apple Watch Series, it was inferred by comparing the date of publication with the Apple Watch Series release dates.

## Results

### Literature Review

The literature search strategy resulted in 5583 paper titles being identified. Screening of titles and abstracts resulted in 2.06% (115/5583) of papers being selected and reviewed in full. Of these 115 papers, 19 (16.5%) were identified as related to Apple Watch validation or comparison studies, which are summarized in Table 1.

**Table 1:** Summary of Apple Watch validation studies (N=19).

Study	Study focus	Outcome
Binsch et al [21], 2016	Resilience and workload monitoring	PPG^a^ reliable in the at-rest condition; wide-ranging outcomes during movement Apple Watch showed the most variance in steps and distances compared with ground truth measurements, followed by the comparison, Fitbit Surge and Microsoft Band Such variances are surmised to be because of differences in data resolution and access and underlying algorithms using accelerometer and GPS data for step count estimation
Shcherbina et al [22], 2017	HR^b^ and EE^c^	Lowest error in HR and EE for cycling; highest error for walking Apple Watch achieved the lowest overall error in HR and EE of the tested devices (Basis Peak, Fitbit Surge, Microsoft Band, Mio Alpha 2, PulseOn, and Samsung Gear S2)
Dooley et al [23], 2017	HR and EE	Apple Watch HR mean absolute percentage error was between 1.14% and 6.70%, not significantly different during baseline and vigorous-intensity treadmill exercise; lower HR in light- or moderate-intensity treadmill exercise and recovery EE mean absolute percentage error was between 14.07% and 210.84%, measuring higher EE in all states compared with the criterion measure (Parvo Medics TrueOne 2400), with greater errors for higher BMI and the male population HR and EE results were mostly better than other tested devices (Fitbit Charge HR and Garmin Forerunner 225)
Wang et al [24], 2017	HR	Apple Watch had 95% differences between −27 bpm^d^ and +29 bpm; concordance correlation coefficient was 0.91; accuracy diminished with exercise.
Hernando et al [25], 2018	HRV^e^	Apple Watch RR interval data were found to contain gaps lasting 6.5 seconds per gap, averaging 5 gaps per recording, not correlated with stress or relaxation case The cause is surmised to be because of failure to detect reliable pulses from PPG data Temporal HRV indices were not significantly affected, but frequency-based LF^f^ and HF^g^ power showed a significant decrease Apple Watch was able to successfully detect mild mental stress
Abt et al [26], 2018	Moderate-intensity exercise	Apple Watch threshold for moderate-intensity exercise was lower than the defined criterion of 40% to 59% VO2R^h^, leading to overestimation of moderate-intensity exercise minutes
Abt et al [27], 2018	Maximal HR	Apple Watch had good to very good criterion validity for measuring maximal HR with no substantial under- or overestimation Moderate and small errors were found for simultaneous recording from left versus right watches
Roomkham et al [28], 2019	Sleep monitoring	Apple Watch raw acceleration data were used to compute ENMO^i^ for classification Apple Watch had high accuracy (97.3%) and sensitivity (99.1%) in detecting sleep and adequate specificity (75.8%) in detecting wakefulness
Perez et al [29], 2019	AF^j^	Apple Watch irregular rhythm notification was triggered on 0.52% of 419,297 participants Of those who returned an ECG^k^ patch, 84% of subsequent notifications were confirmed to be AF A total of 34% of ECG patches returned identified AF in part because of the transient nature, suggesting that Apple Watch may be useful for ongoing monitoring
Nuss et al [30], 2019	EE	Apple Watch overestimated EE in women and underestimated EE in men Pooled relative error was 24.3%, 18.6% for men, and 19.9% for women Neither device showed accurate results compared with EE measured with a MetCart
Thomson et al [31], 2019	HR	ECG correlation was strongest for very light intensity with a >0.90 concordance correlation coefficient Most relative error rates were <5% with a maximum of 5.73% Apple Watch was more accurate in recording HR than the Fitbit Charge HR 2
Nelson and Allen [32], 2019	HR and passive monitoring	Apple Watch 3 was generally accurate across a 24-hour period compared with ECG; the mean difference was −1.8 bpm, the mean absolute error was 5.86%, and the mean agreement was 95% Apple Watch was more accurate than Fitbit Charge 2
Falter et al [33], 2019	HR and EE in patients with cardiovascular disease	Apple Watch showed good correlation without systematic error comparing Apple Watch PPG HR with ECG ground truth Apple Watch showed a systematic overestimation of EE compared with indirect calorimetry Apple Watch HR accuracy was clinically acceptable
Düking et al [34], 2020	HR and EE	Apple Watch 4 showed the highest validity in measuring HR, followed by Polar Vantage V, Garmin Fenix 5, and Fitbit Versa The coefficient of variation for HR was 0.9% to 4.3% and, for EE, it was 13.5% to 27.1%
Espinosa et al [35], 2020	Step counting and HR	The walking error was 2.6%; jogging error was 5.1% HR limit of agreement was −2.2 to 1.8 bpm for walking and −3.5 to 4.3 bpm for jogging Apple Watch displayed a high level of agreement and was highly accurate
Seshadri et al [36], 2020	HR in patients with AF	Patients with AF showed a correlation coefficient of 0.7 between Apple Watch 4 and telemetry Apple Watch 4 HR was more accurate for patients in the AF condition than for those not in the AF condition Caution suggested in Apple Watch HR monitoring in patients with arrhythmia
Seshadri et al [37], 2020	AF	Apple Watch 4 notification correctly identified AF in 34 of 90 instances (41% sensitivity), with no false positives and 31% inconclusive The agreement between Apple Watch 4 and telemetry was 61% Apple Watch–exported ECG PDF files showed AF in 84 of 90 instances (96% sensitivity), no false positives, and 2 failures to generate PDFs Agreement between Apple Watch 4 ECG PDFs and telemetry was 98.9% Further validation is required because of the high inconclusive result rate
Glasheen et al [38], 2021	Wheelchair use	Apple Watch 1 only showed good agreement on higher-rate fixed-frequency tasks, with significant overestimation at low frequency Arm ergometry showed good agreement across all cadences Overground tasks showed poor agreement, with significant differences found
Huynh et al [39], 2021	HR in patients with obstructive sleep apnea and AF	Apple Watch 1 variability increased as the magnitude of the HR measurement increased The Lin concordance correlation coefficient was 0.88, suggesting acceptable agreement between Apple Watch 1 and telemetry

^a^PPG: photoplethysmography.

^b^HR: heart rate.

^c^EE: energy expenditure.

^d^bpm: beats per minute.

^e^HRV: heart rate variability.

^f^LF: low-frequency.

^g^HF: high-frequency.

^h^VO2R: reserve oxygen consumption.

^i^ENMO: Euclidean norm minus one.

^j^AF: atrial fibrillation.

^k^ECG: electrocardiogram.

Several published reviews focusing on wearable devices, smartwatches, and associated physiological measurements were also identified as part of this search (Textbox 2). These reviews provide a contextual background in a number of areas; however, this review was focused on Apple Watch–specific research.

**Wearable device reviews identified.**

**Authors and review focus**

- Lu et al [40], 2016: health care applications
- Reeder and David [41], 2016: health and wellness
- Kim et al [42], 2018: stress and heart rate variability
- Jo et al [2], 2019: patient benefits from wearable devices
- Shin et al [43], 2019: accuracy, adoption, acceptance, and health impact
- Attig and Franke [44], 2020: reasons for abandonment of personal tracking
- Guillodo et al [45], 2020: clinical applications of wearable-based sleep monitoring
- O’Driscoll et al [46], 2020: accuracy of energy expenditure monitoring
- Hickey et al [47], 2021: detect and monitor mental health conditions and stress

### HR and HRV

#### Overview

Across the Apple Watch Series, there are several mechanisms for detecting and monitoring HR metrics. At a minimum, all Apple Watch Series use photoplethysmography (PPG) optical HR sensors to detect either low or high HR and irregular rhythm. In the newer model Apple Watch, there is the option for additional sensors to record ECG. Therefore, Apple Watch users have access to 2 independent measurements of HR through separate apps that can serve similar functions to medical devices [48].

Traditionally, clinical HR and cardiac assessments are performed with 12-lead ECG recordings; however, this is unsuitable for continuous monitoring applications. Wearable devices generally use PPG- and ECG-based sensors, which can be more easily integrated but provide less information. Irregular HR notifications check for events that show irregular rhythm that “may be suggestive of AF” [49]. In Apple Watch Series 1 onward, notifications can be derived from PPG-based tachograms captured opportunistically at irregular times during the day and subsequently classified using an algorithm [50]. In the event that irregular heart activity is detected within the ECG version 2 app, the Apple Watch (Series 4 onward) classifies the ECG recorded event as either atrial fibrillation (AF), sinus rhythm, high or low HR, or inconclusive or declares a poor reading.

The Apple Heart Study, conducted from November 2017 to August 2018, assessed 419,093 enrolled participants via PPG recordings to determine the presence of previously undiagnosed AF [29,50,51]. If an AF event was detected with a duration of >30 seconds, the patient was offered a telemedicine consultation and ePatch ambulatory ECG patch for confirmatory monitoring over a period of up to 7 days. The study noted that of the participants who had been notified by the Apple Watch of the presence of AF, only 34% had subsequent ECG recordings conducted via mailed ECG patches [29]. However, 84% of the app-detected AF notifications were concordant with subsequent clinical AF diagnoses [29].

A pilot validation study monitoring HR via PPG to detect the presence of AF in patients with obstructive sleep apnea found an agreement between the Apple Watch HR–declared events and GE Healthcare CARESCAPE Monitor B650 telemetry [39]. The findings concluded that 95% of the HR readings made by the Apple Watch Series 1 measured within 19 beats per minute (bpm) of telemetry with a Lin concordance correlation coefficient of 0.88 and a mean bias of 0.26 bpm. These values were considered acceptable but relatively wide. Another study used the Apple Watch Series 1 to detect clinical correlations between HR during subacute periods in patients recovering from acute myocardial infarction [52]. HR recordings were taken 4 times per day during a 30-day postdischarge period. Healthy patients showed a decline in average daily HR of 0.2 bpm per day compared with patients with prior coronary artery bypass surgery showing an increasing HR trend of 0.1 bpm per day and those with hypertension and type 2 diabetes mellitus showing a slower HR decline.

A study by Shcherbina et al [22] compared the Apple Watch (presumed to be Series 1) with other commercially available wrist-worn devices. It found that the Apple Watch using the Apple Health app was able to provide HR, EE, and step counts sampled at 1-minute intervals or more frequently if higher-intensity exercise was detected or declared by a workout routine [22]. All other commercially available wrist-worn devices in this study, including the Basis Peak, Fitbit Surge, Microsoft Band, PulseOn, and Samsung Gear S2, only had granularity down to 1 minute. Across all modes of activities, the Apple Watch achieved the lowest error of all tested devices, averaging a 2% error in HR. This was echoed in another 11% (2/19) of studies comparing the accuracy of HR within Apple Watch devices with other commercially available devices relative to traditional ECG [23,33].

Derived from HR is HRV, another measurement of cardiac performance indicating the variation in time between heartbeats (NN or RR interval) in either the time or frequency domain. It is a method for monitoring cardiac health, sleep quality, mental stress, chronic pain, posttraumatic stress disorder, bipolar disorder, and traumatic brain injury [53,54]. There are a number of statistical methods to calculate HRV, including the SD of NN intervals (SDNN), the HRV triangular index, the SD of the average NN intervals, and the root mean square of successive differences [55,56]. The Apple Watch provides HRV as the SD of the beat-to-beat intervals (SDNN) [57]. Although HRV can be calculated from ECG, in the case of the Apple Watch, it is calculated using the optical HR sensors and can be accessed within HealthKit on a paired iPhone device.

Dalmeida et al [58] looked at HRV features in the time domain and the high- and low-frequency domains to determine the most ideal metric by implementing a machine learning algorithm. They concluded that SDNN, as used by Apple Watch, was acceptable among other methods for calculating HRV [58]. The Apple Watch data used with the developed web application for this study predicted stress states with 71% probability and relaxation states with 79% probability. Another validation study by Hernando et al [25] investigated the impacts of various HRV statistical models on both the time and frequency domains in both relaxed and stressed states and compared the various statistical methods for their accuracy. Approximately 10% of beats were missed, usually consecutively, with a greater number of missing beats in the stressed state and at the beginning of recordings. This is speculated to be because of poor skin contact or sudden movement; however, no empirical evidence is available because of the proprietary nature of the algorithms within the Apple Watch. Computed time domain HRV metrics were comparable with data from a Polar H7 chest belt, with frequency domain metrics showing differences because of the missed beats [25]. It was found that there was no significant difference in the effectiveness of time domain HRV methods and that SDNN was just as effective as other methods.

#### Applications in Mental Health

The potential of wearable devices for monitoring mental health and related physiological stressors lies in the prospective ability of users to interpret and understand their emotional awareness and emotional regulation or of this information to be collected and relayed to a caregiver or clinician for follow-up action.

Panic disorders commonly present with other mental health issues, for which monitoring can prove to be valuable. Panic attacks are specified as sudden or abrupt surges of involuntary arousal, increasing HR rapidly and subsiding within minutes, and are commonly preceded by cardiorespiratory instabilities [59]. These involuntary movements are controlled by the autonomic nervous system, which is part of the peripheral nervous system. The autonomic system comprises sympathetic and parasympathetic systems that have significant control over HR, HRV, blood pressure, respiration rate, and temperature [60]. In simple terms, sympathetic activity leads to arousal or “fight or flight” responses, whereas parasympathetic activation leads to more recovery activity. Research on the psychological significance of the imbalance between these 2 systems suggests that HRV could be used as a more ideal physiological measurement of stress compared with HR. Reduced HRV is seen in individuals with psychiatric disorders [61]. This is because low-frequency components of HRV indicate increased sympathetic activity, whereas high-frequency components are generated within the parasympathetic system. An imbalanced ratio between low- and high-frequency components suggests a greater presence of stressing stimuli [42,58]. These findings were also encouraged by a systematic review of wearable devices, which determined that HRV was “the most useful metric for detection of stress and anxiety” and that devices that combined accelerometers, ECG, and subjective questionnaires could assist in the diagnosis of depression [47].

Physiological data accuracy with regard to HR and HRV is generally viewed as favorable compared with other devices, especially in the at-rest condition, and is likely to provide valuable data for the needs of mental health monitoring applications.

### EE Measurement

#### Overview

Another key tracking feature is step counting and the average or total calories burned through EE. A key feature of EE and movement tracking is the motivation provided by setting personal activity goals. The Workout app used for the Apple Watch assists in tracking progress updates and setting activity goals. Motivation goal setting can assist in weight management and overall health tracking and can be programmed within the Apple Watch [62]. Apple provides several apps that can be used with the Apple Watch to assist in health tracking and statistical data collection with the Workout and Activity apps. The Workout app includes a list of activities (Table 2), an automatic workout detection feature, a record of workout sessions (including start and end times), progress update tracking, and reminders to start routines. The Activity app is used to monitor general activity and movement throughout the day and is intended to encourage users to move, stand up, and exercise. Activity targets are displayed using dynamically closing rings, illustrating a clear overall goal [63]. Passive data such as HR, steps, distance, active minutes, and stand reminders are collected. The total EE calculated from the Apple Watch accelerometer was noted to improve with the inclusion of HR in the calculation algorithm [46,64]. As such, the Apple Watch continuously measures HR in the Workout app during exercise and for 3 minutes afterward to calculate a “recovery rate,” which is further used to enhance the estimate of how many calories have been burned during the workout routine [48].

Wearable devices are typically able to determine the difference between low- and high-intensity activity but require improvement in resilience to changes in setting, particularly with an increase in exercise intensity, if more accurate absolute EE is to be extracted. Most validation studies that included the Apple Watch indicated an overestimation of total EE at different activity intensity levels [26,33,38,65]. However, 11% (2/19) of the studies noted an underestimation of total EE in the study group, and 5% (1/19) of the studies noted that the Apple Watch overestimated EE in female participants but underestimated it in male participants [30,64]. Despite the variation in the accuracy of EE estimation, the device could successfully distinguish activity intensity. This is summarized in a systematic review of activity trackers and total EE proficiency by O’Driscoll et al [46], which noted that devices exhibiting the largest EE error relied exclusively on accelerometer data.

At present, a range of activity types and intensities can be defined by the wearer (Table 2) [66]. This would enable the Apple Watch to generate an improved EE estimate [52]. Additional data, such as altimeter data to indicate changes in elevation, could further improve this estimate. Modifications to the accuracy of algorithms for activity tracking and calorie counting can be improved with software updates and more nuanced user input; for example, watchOS 8 (released in September 2021) adds outdoor cycling detection, e-bike pairing for improved calorie calculations, and Pilates and tai chi workout types [66,67].

**Table 2:** Workout types for Apple Watch within the Workout app.

Activity type	Subtype	Notes
Walking	Indoor or outdoor	Apple Watch Series 1 requires iPhone to calibrate pace and distance calculated from GPS (Apple Watch Series 2 onward) Elevation from altimeter (Apple Watch Series 3 onward)
Running	Indoor or outdoor	Option to use Bluetooth chest strap instead of integrated PPG^a^ heart sensor to reduce motion artifacts
Cycling	Indoor or outdoor; e-bike or manual (watchOS 8)	Speed and distance (Apple Watch Series 2 onward) and map elevation (Apple Watch Series 3 onward) Automatic detection for start and stop (from watchOS 8)
Elliptical	Elliptical machine	N/A^b^
Rower	Rower machine	N/A
Stair stepper	Stepping machine	N/A
HIIT^c^	Intense exercise followed by short periods of rest (30-45 seconds)	May affect HR^d^ sensors Calories tracked with accelerometer
Hiking	Tracks pace, distance, elevation gain, and calories burned	Requires altimeter (Apple Watch Series 3 onward) or paired the phone with an altimeter
Yoga	All types of yoga	N/A
Functional strength training	Dynamic strength training with dumbbells, resistance bands, and medicine balls	N/A
Dance	All types of dance	N/A
Cooldown	Easy moves and stretches	N/A
Core training	Strength-building of abdominals and back	N/A
Swimming	Pool or open swim	Set pool length; GPS is not used to conserve battery Open swim requires GPS; may affect HR sensors
Wheelchair	Outdoor wheel-walk pace and outdoor wheel-run pace	Apple Watch Series 2 onward uses GPS or paired iPhone with GPS for Apple Watch Series 1 Measures time, pace, distance, calories, HR, and pushes
Other	Add a workout type	HR and motion sensors work together to provide an accurate reading Will display popular workouts from users

^a^PPG: photoplethysmography.

^b^N/A: not applicable.

^c^HIIT: high-intensity interval training.

^d^HR: heart rate.

#### Applications in Mental Health

Personal activity tracking and goal setting can lead to increased exercise, with physical and mental health benefits. The key components of mental health benefits can be seen in individualized means of self-reflectivity and mindfulness [15]. Tracking changes in activity and movement can be used as an indicator of health management, such as weight loss, but also as a key indicator of changes in mood stages (eg, low activity could indicate the presence of a depressive episode). A cross-sectional study investigated the effects of wearable trackers and how they make users feel and concluded that most users felt positive about tracking technology and that negative experiences were mostly confined to individuals with low conscientiousness or openness to experience [68]. Further investigation of wearable trackers and their psychological effects in younger demographics is recommended, as well as an examination of the effects in those who exhibit neuroticism and obsessive-compulsive traits [68].

There is some ambiguity regarding the level of accuracy that is acceptable for EE, as it depends on the context of the application. For wellness applications, the absolute accuracy of EE may not be critical or align with the primary goal of the intervention. In this case, small inaccuracies may not be particularly significant for the user. Tracking of general movement patterns in combination with measures of HRV and respiratory rate variability may be sufficient for monitoring work-related stress, detecting episodes of mania, anxiety or depression, or sleep-related disorders (insomnia) [69,70]. Similarly, the detection of psychological distress through activity metrics appears viable [71]. However, more research is required to validate the capability of the Apple Watch to detect such episodes.

### Sleep Monitoring

#### Overview

The introduction of watchOS 7 in June 2020 brought about integrated sleep monitoring to track the quality and duration of wearers’ sleep for Apple Watch Series 3 and above. The watchOS 8 release in September 2021 improved this by also reporting sleeping respiratory rate [72]. As this is a relatively recently introduced feature, which is primarily promoted as a “wellness monitoring” feature, no literature was identified that tested or validated it. Sleep tracking through third-party apps is also available, some of which are more sophisticated and integrate HR measurements from PPG [73].

Roomkham et al [28] performed a 27-night sleep study with the Apple Watch Series 1 using raw data from its accelerometers at 50 Hz through Apple’s Core Motion framework (independent from the watchOS Sleep app, which did not exist at the time) and compared the results with the Philips Actiwatch Spectrum PRO [28]. The overall patterns between the 2 devices demonstrated correlations of key movement events with 97.3% accuracy and 99.1% sensitivity in detecting sleep and a specificity of 75.8% for detecting wakefulness.

However, wrist-worn sleep monitors based on accelerometry are not without criticism, and there is some skepticism about the reliability of using wrist-worn devices for monitoring sleep to identify the depth of sleep and wake periods. Approximately 5% (1/19) of the studies looked into 3 devices—the Mi Band activity tracker, the MotionWatch 8, and the Sleep Cycle mobile phone app—to monitor sleep [74]. All devices reported high accuracy of time in bed but were incapable of accurately detecting sleep and wake periods and sleep efficiency. This study also found that each of the devices had unacceptable levels of agreement with polysomnography. This view was echoed in a systematic review of wearable devices for sleep monitoring, which stated that wearables generally have “acceptable sleep monitoring but with poor reliability” [45]. It is evident from these studies that using wrist-worn accelerometers as the sole sleep-monitoring sensor severely limits the ability to contextualize sleep patterns and behavior. As such, they are not capable of full-spectrum sleep monitoring but remain promising.

#### Applications in Mental Health

It is recognized that low quality of sleep may exacerbate physical and mental health problems and that sleep tracking can be used to improve user awareness of possible sleep problems [75]. The prevalence of insomnia and chronic sleep issues such as sleep apnea is increasing, with an estimate that 1 in 2 people experience bouts of sleep disturbances during their life, with negative impacts [39,45]. Sleep monitoring is also valuable for mental health monitoring, as a lack of sleep can be the cause of impaired performance, low energy levels, and problems with mood.

The literature indicates that most wearable devices with accelerometers have high sensitivity but low specificity for sleep detection [45]. Specific information about the quality of sleep would require other sensor data or could be inferred through patient-practitioner communication. However, there are practical concerns regarding battery use and when the device can be charged, as many users may prefer to charge their Apple Watch devices overnight [76]. Charging creates interruptions in monitoring, which could pose a challenge in accurately monitoring panic attacks, which usually occur unexpectedly [28,59,77]. Improvements in charging times have occurred with the announcement of Series 7, which includes the Apple Watch Magnetic Fast Charging USB-C cable that can charge to 80% battery capacity within 45 minutes, which may serve to minimize such interruptions [78]. Limitations in the accuracy and detail of sleep quality restrict clinical utility in cases of mood disorders, mania, anxiety or panic attacks, and sleep-wake disorders, which may require investigation in a specific sleep cycle. The interpretation of sleep data can be complicated by incorrect sleep detection (eg, while being still or watching television) [75]. However, in combination with other tools and strategies, general sleep monitoring and tracking can assist in developing and implementing behavior change techniques.

## Discussion

### Apple Watch Sensors

**Figure 1:**
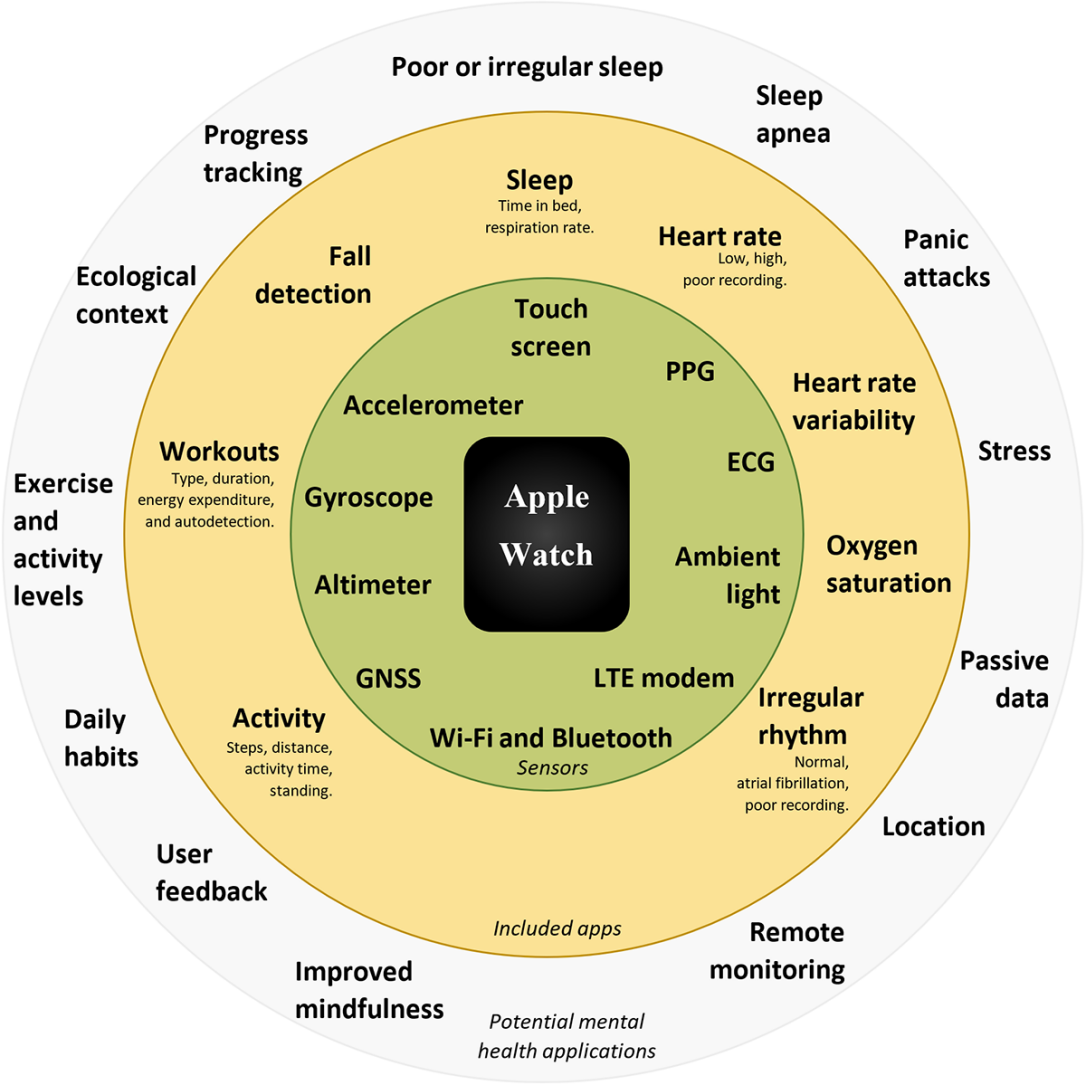
Summary of Apple Watch sensors, apps, and potential mental health applications. ECG: electrocardiogram; GNSS: global navigation satellite system; LTE: Long-Term Evolution; PPG: photoplethysmography.

The Apple Watch is a sensor-rich, well-constructed, and connected device. It uses a large range of apps and has significant potential for applications in mental health (Figure 1).

**Figure 2:**
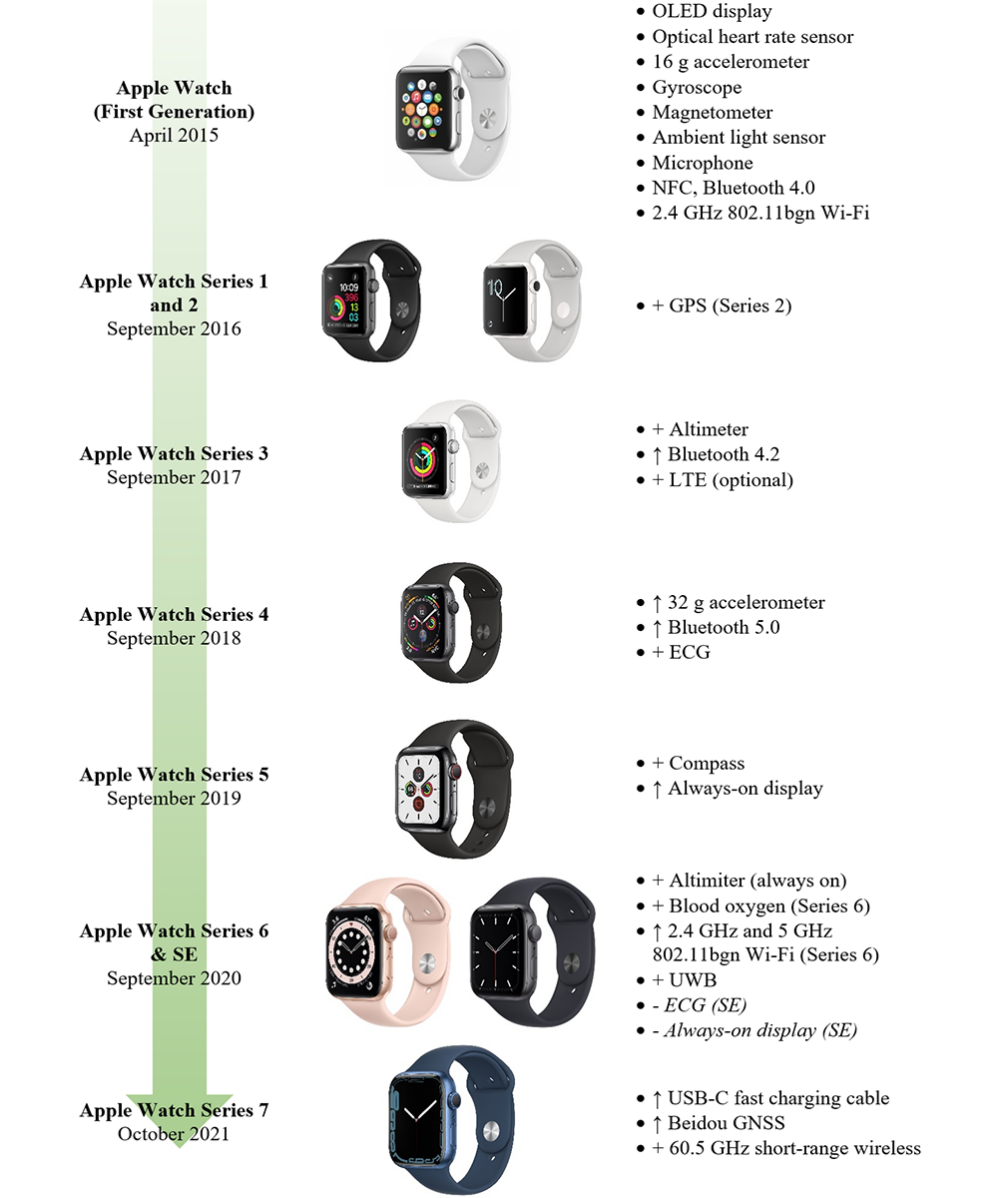
Evolution of Apple Watch Series features. Feature upgrades (↑) and new feature additions (+) are indicated. ECG: electrocardiogram; GNSS: global navigation satellite system; LTE: Long-Term Evolution; NFC: near-field communication; OLED: organic light-emitting diode; UWB: ultrawide band.

Apple Watch sensors typically include a 3-axis accelerometer, a gyroscope and magnetometer, optical PPG-based HR sensors, altimeters, ambient light sensors, temperature sensors, ECG, and capacitive (touch) sensors [3]. Across each iteration of the Apple Watch, sensor inclusions and capabilities have increased, matched with software updates aimed at increasing the overall accuracy of the collected data. Figure 2 presents a timeline of the development of the Apple Watch, summarizing the changes in sensor inclusions over time. The latest version of watchOS (version 8.0.0) is supported by Series 3 to Series 7 models. The models currently available for purchase include Series 3, SE, and Series 7. The Apple Watch Series 3 does not include fall detection as the 6-axis inertial measurement unit containing the gyroscope and accelerometer was modified for later-generation Apple Watches [49].

One of the primary sensors in all generations of the Apple Watch is the optical HR sensor, which is used to collect HR data. The scientific principle that these sensors rely on is PPG to detect the amount of blood that is flowing through the wearer’s wrist at any given moment. The reflection of green and infrared light-emitting diode (LED) light is measured with photodiodes that allow for the determination of HR as a periodic variation in the signal. By flashing hundreds of times per second, the optical HR sensor can measure HR across a range of 30 to 210 bpm [48]. Infrared light is used to measure HR in the background and for HR notification systems as infrared light can penetrate the skin better; however, this makes it more susceptible to motion artifacts. Green LEDs are used for workouts and to calculate HRV [48]. The Apple Watch will automatically detect when there is an increase or decrease in motion from the inertial measurement unit and change the LED light color accordingly. Variations have been made in the design and layout of the LED and photodiode arrays with each iteration of the Apple Watch to improve accuracy [79]. These optical HR sensors are used by the Irregular Rhythm Notification Feature (IRNF), which can assist in the detection of AF [80,81]. A red LED was added in Series 6, enabling blood oxygen saturation calculation by comparing the ratio of infrared light and red light. Reflectance oximetry is noted as being less accurate than clinically used transmittance oximetry [79], and we did not identify any literature validating the accuracy of the Apple Watch blood oximetry.

In addition to the optical HR sensors, from Series 4 onward (not including the SE model), an ECG electrode was integrated into the back face of the watch and the digital crown. When engaged by the user’s finger, a closed circuit is created to measure the electrical potential across the heart, similar to a 1-lead ECG. An ECG measurement takes 30 seconds. The ECG sensor is exclusively used with the ECG classifier to categorize heart events as AF, normal sinus rhythm, high or low HR, or inconclusive [48,82]. Version 2 of the ECG app also includes additional classifications of AF, high HR, and poor recording. For the earlier Apple Watch Series, a third-party accessory (Kardia Band) could be used to provide a 1-lead ECG that achieved a sensitivity of 93% and a specificity of 84% when compared with a standard tachograph [83].

A clinical study compared the ECG app developed by Apple Inc with an FDA-cleared clinical ECG device (GE Healthcare CardioSoft ECG device), with recordings verified by 3 independent board-certified American cardiologists in each of the ECG app categories [50]. The app received clearance by the FDA as a De Novo class II device as it was proven to perform similarly to the comparator device [82]. The same approval was also given to the optical HR sensor IRNF software in 2018 [80]. Some limitations exist in the use of both apps, which are not intended to be used on persons aged <22 years. Depending on the country in which the Apple Watch user resides, they may not have access to the software and, as such, may not be able to use these notification features. In Australia, both the ECG app (version 2.0) and the IRNF software were approved by the Therapeutic Goods Administration of the Australian Government in early 2021 [84,85].

### Further Considerations

Health data collected from the Apple Watch could complement smartphone data collection and self-reported measures to provide additional context and assist in determining and tracking a user’s affective and emotional health. Advancements in the sensing technologies available within wearable devices and enhanced user interfaces have removed some of the previously limiting factors of monitoring mental health using wearable technology. However, the current general consensus for using wearable device sensors is that they should be paired with traditional screening and diagnostic tools and not be considered as a replacement [33,83]. Wearable devices can assist in clinical diagnosis and application of therapy if the findings are consistent with the patient’s complaints or concerns or if the patient is unsure of their physiological level of stress [86]. Indeed, a systematic review of digital health interventions for depression and anxiety in young people has shown that such interventions may only be of clinical significance when their use is highly supervised [87].

An article compared several wearable devices, including the Apple Watch (series unspecified), and their applications for “advancing resilience and mental health of employees that experience high workload” [21]. The study noted that an increase in psychological disabilities in the modern workplace requires the development of new and emerging technologies to measure and monitor physical or mental status. As such, these tools are being implemented to assist in the diagnosis and treatment of stress within professional workplaces and in a performance review. A potential issue with workplace inclusion for monitoring mental health and wellness is regulations and access to technology.

The use of the Apple Watch as a source of data may address problems with patient recall bias as most assessments are reliant on patient self-reporting. This could reduce the reliance on patient memory and continued questioning to ensure consistency. In addition, it could be a relatively low-cost method for better long-term tracking of symptoms and trends in the data [69]. The use of these data permits the construction of an ecological context that could empower a more cohesive diagnosis and application of therapy or assist in refining threshold values used in algorithms toward a validated measure.

Although there are potentially great benefits of wearable devices in improving mental health, there are some potential drawbacks, including concerns about abandonment rates. Approximately 11% (2/19) of individual studies commented on the long-term use of electronic wearables, one noting that 20% of consumers stop using their wearables after 3 months, and <50% continue to use them after 1.5 years [83,88]. This is compounded by the need to provide enough contextual information regarding the data collected, which requires some level of active user participation. For a clinical diagnosis of a mental disorder, clinicians must make a decision based on weighing the mix of potentially contradictory evidence according to their expert judgment, which could require symptom tracking over a period of months to come to a clear conclusion. Symptom tracking for the validation of several mental health diagnoses against the Diagnostic and Statistical Manual of Mental Disorders can require the presence of symptoms over a period of weeks, months, or even years for mood disorders, anxiety disorders, and schizophrenia [59].

A validation study was completed on the effectiveness of using the Apple Watch to collect passive sensor data with “ecological momentary assessments” from a watch-based questionnaire app recording patient feedback to assess and monitor substance abuse in young adults [89]. The response from participants on the perceived burden of engaging with the app was low; however, it was noted that the relative ease of completing the surveys was easier on an iPhone than on the Apple Watch. Burdensome interactions within wearable devices could reduce uptake and willingness to use technology for mental health monitoring. However, the benefits of engaging users through health notifications and alerts can assist in seeking medical assistance or outpatient care [29]. A longitudinal observational study using cognitive assessment delivered through the Apple Watch in patients with major depressive disorder noted excellent adherence for both mood and cognitive tests (95% and 96%, respectively) over the 6-week study period, and it was not influenced by symptom severity or cognitive function at the study onset and did not deteriorate over time, supporting the feasibility of this approach [90].

### Health and Sensor Data Access

The availability of sensor and health data collected from the Apple Watch and patient input relies on the application programming interface frameworks available from Apple for iOS and watchOS. The main frameworks are HealthKit, ResearchKit, CareKit, and SensorKit [91-93]. HealthKit is the most comprehensive as it implements a central repository for all collected health data related to the user. Developers can write apps that request permission to access the HealthKit data store to record, access, and share user health data. SensorKit is used in the event that raw access to sensors is required. ResearchKit may be used to build research study apps, whereas the CareKit framework is suited to the development of ongoing care capabilities. Together, these frameworks allow for the implementation of apps that can collect raw data and store and analyze collected data (including passively collected data) and provide tracking feedback to the end user as well as the clinician.

Within the HealthKit framework, a range of rigid data classes and methods can be used to collect, store, and retrieve data. In this way, virtually all types of health-related data can be stored as numerical data (eg, HR) and categorical data objects (eg, blood type). It categorizes the data systematically, reducing duplication and allowing for straightforward statistical data analysis. HealthKit supports units of measurement within each of these categories such as length, mass, volume, and energy. Conversion between measurement systems is automatically supported within the framework but can also be explicitly defined. Developers cannot create custom data types or units but can use the metadata fields to store additional data.

Most of the identified studies investigating wearable devices collected the activity level (steps and caloric expenditure), HR, and sleep data without indicating how the data were collected from the device, the frequency of data recording, or which measures were extracted from HealthKit. We believe this to be important information to be provided by studies, especially those that develop a custom app, to ensure a comprehensive understanding of the data, allow for comparative analysis with other studies, and inform future developments.

### Data Analysis and Digital Phenotyping Approaches

Digital phenotyping approaches have been an active area of development enabled by the popularity of smartphones [94]. By collecting data from sensors in a smartphone on a moment-by-moment basis, it is hoped that information about the user’s behaviors can be inferred to personalize patient care [95]. Active and passive data collection techniques have been explored, including data such as location, activity, app use, phone use, Bluetooth signals, and voice samples [96]. Research has focused on correlating such data with reported and diagnosed conditions to determine the most valid signals for mental health applications; however, this is still considered to be in its infancy.

Early studies suggest that data surrounding activity and geolocation could serve as early signs of mania or depression [97]. Furthermore, the monitoring of movement and light data was able to detect and assess depression severity [98]. Research into schizophrenia shows that digital phenotyping approaches have merit in identifying relapse events [99], that collected accelerometer and GPS data have a good correlation with future patient survey scores [100], and that such an approach was tolerated by outpatients [101].

Issues surrounding noise, privacy preservation, missing data, and data quality have been acknowledged and pose challenges in data analysis as the sensors may not be able to provide a complete context [102]. However, such approaches still require considerations of clinical relevance, social equity, development of common data standards, and multidisciplinary collaboration [103,104]. This may include the need to improve digital health literacy through training programs tailored to the needs of the target population [105].

Although it may be theoretically possible to combine smartwatch data with those collected from a smartphone to improve data quality for digital phenotyping approaches, as a smartwatch is more likely to be worn on the body than to be left behind, such an approach may be incompatible with smartwatches, which are much more resource constrained in terms of computational power, storage, connectivity, and (most importantly) battery power. The continuous collection of sensor data on smartphones has been shown to have a significant impact on battery life, which is a factor against user acceptance [103]. The impact on smartwatches, which typically have smaller batteries and rely extensively on sleep power-saving techniques to achieve all-day battery life, is anticipated to be significant.

As a result, it seems most prudent to identify the relevant physiological and physiologically related signals that relate to mental health and build algorithms focusing on data from those metrics alone rather than taking a dragnet correlation approach as is traditionally used in digital phenotyping. Such an approach will also serve to address some of the concerns regarding privacy and user perceptions that such a system is fated to diagnose users with conditions simply based on overcollection of data and misunderstanding of cause and effect [106].

### Personal Health Information

The issue of personal health information regulation is important for maintaining user trust and privacy. Regulations have usually lagged behind rapid technology development, with concerns about data ownership. As such, there is some suggestion that wearable technology be considered differently from consumer technology because of inherent personal health information concerns.

Consumer wellness devices are not considered medical devices and, thus, may not be as accurate or reliable for remote health monitoring. Establishing their accuracy would require independent verification or undergoing regulatory approval processes. Constraints surrounding medical device regulation are a source of concern as the long process can stifle innovation and the development of new technologies [107]. However, some features may be able to individually receive clearance from regulators (eg, the ECG app with the Apple Watch) [108]. The ECG app and IRNF are both classified as De Novo within the FDA regulations, which is a marketing pathway for novel devices of low to moderate risk where a predicate device does not exist. In this manner, the FDA creates a classification for the device, which can be used for future premarket approvals of equivalent devices to ensure that new and emerging novel technologies are not held back during classification.

In addition, most device manufacturers provide their own independent platforms, very similar to HealthKit for the Apple Watch, for users’ data storage. These platforms may be limited in terms of data access and sharing, forming a vendor lock-in that prevents users from being able to migrate their personal health information to other platforms and reducing the research value of the devices. There are concerns over the control larger companies may have over the health data of users; this can conflict with informed consent, which is integral to medical practice [69]. Passive data collection is less intrusive and time consuming for the wearer; however, it can capture a large amount of personal data that can be stored unknown to the user, even if they have authorized the data to be recorded. Typically, the average person is more relaxed with security implementation when using personal devices and may be unaware of the level of security that third-party apps provide [13]. Similar concerns surround wearable devices and their use in workplace wellness programs and health insurance provisions if there is ambiguity regarding how the data will be used and the potential for surveillance [13,14]. The ethos behind the Apple HealthKit framework’s rigid type structures and fine-grained authorization process is designed to ensure that only necessary data are collected or accessed [109,110].

The use of wearable technology for health care service provision is still in its infancy, and evidence to support its implementation is still being developed. Known concerns exist regarding passive data collection, data ownership, data use, user trust, and user attitudes toward wearable technologies, leading to potentially high abandonment rates [44,103].

### Current Applications

Perhaps the best model for how the Apple Watch can be applied to mental health can be found in the insurance sector, where some insurance providers have embraced wearable technologies to promote healthier lifestyles. Incentive programs involving wearable devices have been used by numerous US health insurance providers, including United Health Care, Anthem, Humana, Health Care Service Corporation, Centene, CVS Health (Aetna), WellCare, Kaiser Permanente, GuideWell, and Molina [17]. AIA Insurance Australia has a specific program using the Apple Watch called the Vitality Apple Watch Benefit, which reduces the monthly loan repayment of the device through the achievement of weekly activity targets [16]. Loss-framed incentivized policies using the Apple Watch achieve a 34% increase in tracked activity days over 1 month in comparison with a standard gain-framed policy [12]. This offers a potential solution to individuals who may not have the financial flexibility to pay the full upfront cost of the Apple Watch device but can still have access to the benefits of the device as a wellness monitor for personal health. Another study investigated the “incentivize and persuade” health-tracking approach from both insurers and employers for enhancing business chain value. It was concluded that persuaded self-tracking, whereby service firms or employers encourage consumers and employees to collect and share data via self-tracking, is heavily influenced by service firm and individual determinants. Understanding consumer perceptions and consumer reactions within a conceptual framework should reflect values in use, privacy and security, and perceived fairness or justice as the technology itself may perpetuate inequalities [15]. Both studies noted the effects of physical activity on physical wellness, as well as mental health, but did not specifically note the impact on policy holders with severe mental illnesses. Investigation into mental health monitoring for insurance purposes could potentially create contention and the consensus that balancing privacy and confidentiality is critical for engendering trust with users and policy holders through transparency [111].

### Conclusions

The Apple Watch has presented itself as a capable wearable device that is able to monitor several physiological parameters and track overall health and wellness. Its use within the mental health sphere is encouraging, particularly as more research emerges correlating changes in the emotional and physiological states of the body. Measures of HRV are key indicators of changes in both physical and emotional states. In combination with other sensors to monitor general activity, sleep, and more, health data can be aggregated with user-provided information to assist in the monitoring and even diagnosis of mental health disorders. Particular benefits may be derived through the avoidance of recall bias by providing a more objective, data-driven record of events in a passive manner. The lack of methodologically robust and replicated evidence of user benefits and a supportive health economic analysis, as well as concerns about storage, access, and security of personal health information, remain key factors that must be addressed to enable broader uptake for mental health applications.

## Abbreviations

AF: atrial fibrillation
bpm: beats per minute
ECG: electrocardiogram
EE: energy expenditure
FDA: Food and Drug Administration
HR: heart rate
HRV: heart rate variability
IRNF: Irregular Rhythm Notification Feature
LED: light-emitting diode
PPG: photoplethysmography
SDNN: SD of NN intervals
